# Volumetric macromolecule identification in cryo-electron tomograms using capsule networks

**DOI:** 10.1186/s12859-022-04901-w

**Published:** 2022-08-30

**Authors:** Noushin Hajarolasvadi, Vikram Sunkara, Sagar Khavnekar, Florian Beck, Robert Brandt, Daniel Baum

**Affiliations:** 1 Department of Visual and Data-Centric Computing, Zuse Institute Berlin, Takustraße 7, 14195 Berlin, Germany; 2 Department of CryoEM Technology, Max Planck Institute of Biochemistry, Am Klopferspitz 18, 82152 Martinsried, Germany; 3 Materials and Structural Analysis, Thermo Fisher Scientific, Takustraße 7, 14195 Berlin, Germany

**Keywords:** Biomedical imaging, Cellular cryo-electron tomography, Macromolecule identification, Deep learning, Capsule network

## Abstract

**Background:**

Despite recent advances in cellular cryo-electron tomography (CET), developing automated tools for macromolecule identification in submolecular resolution remains challenging due to the lack of annotated data and high structural complexities. To date, the extent of the deep learning methods constructed for this problem is limited to conventional Convolutional Neural Networks (CNNs). Identifying macromolecules of different types and sizes is a tedious and time-consuming task. In this paper, we employ a capsule-based architecture to automate the task of macromolecule identification, that we refer to as 3D-UCaps. In particular, the architecture is composed of three components: feature extractor, capsule encoder, and CNN decoder. The feature extractor converts voxel intensities of input sub-tomograms to activities of local features. The encoder is a 3D Capsule Network (CapsNet) that takes local features to generate a low-dimensional representation of the input. Then, a 3D CNN decoder reconstructs the sub-tomograms from the given representation by upsampling.

**Results:**

We performed binary and multi-class localization and identification tasks on synthetic and experimental data. We observed that the 3D-UNet and the 3D-UCaps had an $F_1-$score mostly above 60% and 70%, respectively, on the test data. In both network architectures, we observed degradation of at least 40% in the $F_1$-score when identifying very small particles (PDB entry 3GL1) compared to a large particle (PDB entry 4D8Q). In the multi-class identification task of experimental data, 3D-UCaps had an $F_1$-score of 91% on the test data in contrast to 64% of the 3D-UNet. The better $F_1$-score of 3D-UCaps compared to 3D-UNet is obtained by a higher precision score. We speculate this to be due to the capsule network employed in the encoder. To study the effect of the CapsNet-based encoder architecture further, we performed an ablation study and perceived that the $F_1$-score is boosted as network depth is increased which is in contrast to the previously reported results for the 3D-UNet. To present a reproducible work, source code, trained models, data as well as visualization results are made publicly available.

**Conclusion:**

Quantitative and qualitative results show that 3D-UCaps successfully perform various downstream tasks including identification and localization of macromolecules and can at least compete with CNN architectures for this task. Given that the capsule layers extract both the existence probability and the orientation of the molecules, this architecture has the potential to lead to representations of the data that are better interpretable than those of 3D-UNet.

**Supplementary Information:**

The online version contains supplementary material available at 10.1186/s12859-022-04901-w.

## Introduction

**Fig. 1:**
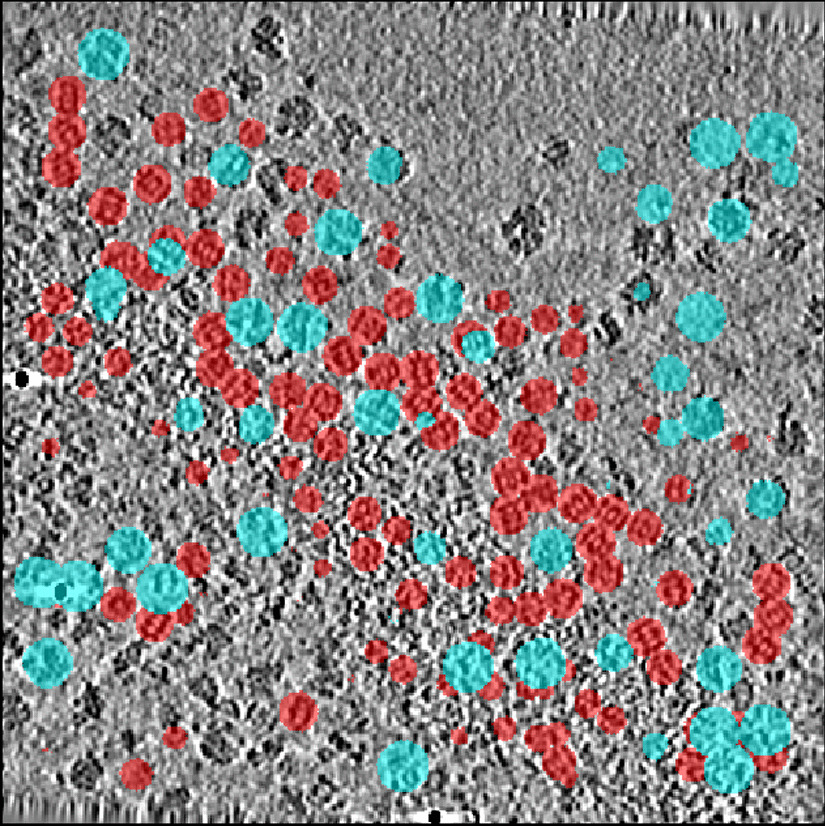
Visualization of macromolecule localization for proteasomes and ribosomes in a sample tomogram of real data set

Understanding biological processes inside a single cell requires detailed knowledge of native structures and the spatial distribution of macromolecular complexes. Although recent advances in cellular cryo-electron tomography (CET) enable researchers to perform 3D visualization of such complex structures in sub-molecular resolution and close to their native state, a lack of annotated data hinders them from developing automated segmentation tools for these structures. Localizing and identifying macromolecular structures in crowded cell environments using fully automated approaches like deep learning (DL) models is not fully explored. Figure 1 illustrates the problem of identifying such complex structures. In essence, most of the proposed architectures [1–3] are limited to Convolutional Neural Networks (CNN) requiring large-scale training data sets annotated by time-consuming user interventions.

Currently the standard method for macromolecule (particle) localization and identification is template matching (TM) [4]. In TM the whole tomogram is scanned using a template density map of the desired molecule and a cross-correlation score is computed for every voxel in the volume. The most highly ranked cross-correlation scores are considered possible particle locations on which sub-volumes can be extracted for downstream tasks like classification and sub-tomogram averaging. The drawbacks of template matching include high computational complexity and difficulties in identification of particles with similar structure.

Various research areas in computer vision like medical imaging have been revolutionized by DL approaches. Recently, several fully automated 2D approaches were proposed for macromolecule identification by different CNN architectures. The first proposed method, DeepPicker, is a 2D CNN with seven layers [5]. The approach includes four pre-processing steps that score, clean, filter, and sort the 2D micrographs. Then, the CNN model is trained using the micrographs and the results of the network are passed through the aforementioned four-step pre-processing stage again. Later, DeepCryoPicker [3] was proposed that has thirteen layers and is trained using small sub-regions extracted from 2D micrographs. In post processing, the sub-regions are stitched together to generate the complete micrograph. Other supervised algorithms include DeepEM [6], Topaz [7] and crYOLO [8]. The former uses the positive-unlabeled learning technique to train a 2D CNN for detecting non-globular asymmetric particles. All these previous models were developed for single particle analysis where the purified proteins are imaged with the transmission electron microscope, resulting in a large number of 2D images each containing thousands of particles. In CET, however, possible target particles need to be identified in the crowded 3D environment of real cells and from a limited number of tomograms.

In a recent work, Moebel et al. proposed the DeepFinder model [2], which employs the 3D-UNet architecture [9] for macromolecule identification in CET images. The model is trained using 3D sub-volumes of the tomograms centered on individual molecules. To validate their model the authors used the synthetic SHREC’19 data set [10] with 10 tomograms plus three experimental data sets consisting of 50, 4, and 5 tomograms, respectively. They report that the model performs better for multi-class identification scenarios than for binary classification. Similar to previous research works [1, 3], they study the $F_{1}$ score evolution with respect to the number of particles in the training set and report that the performance degrades when the number of annotated particles in the tomograms is too low. Also, it is pointed out that binary identification of macromolecules using a limited data size is challenging. Other 3D macromolecule identification algorithms include the one developed by Che et al. [1] based on the Deep Small Receptive Field [11] and CB3D [12] models. CNN architectures are known to be data-hungry. Despite the breakthroughs in data acquisition techniques, preparing a CET data set with a reasonable number of particles as samples to adjust the network weights has remained a bottleneck in this field mainly due to the time-consuming process of annotation.

Motivated by the promising previous results [1, 2], we employ a new 3D DL-based architecture based on CapsNet [13], that is known to be data-efficient [14, 15]. The 3D version of CapsNet was proposed by Zhao et al. [16]. Later, Nguyen et al. [17] replaced the encoder section of the 3D-UNet with a 5-layer 3D CapsNet to perform MRI image segmentation. This model is called 3D-UCaps and has three main components: a feature extractor, an encoder with five layers of 3D capsules, and a decoder consisting of five layers of 3D convolutions. Here, we modify the 3D-UCaps structure to perform macromolecule identification and segmentation. Our experiments and visualization results show that the model performs successfully in both binary and multi-class macro-molecule identification by learning general features like texture and geometrical shapes of the particles extracted by the encoder.

The main contributions of this study are two-fold: First, a deeper neural network architecture is introduced that successfully performs multi-class and binary molecule identification on the test data. Second, a CapsNet architecture is used to improve multi-class and binary classification when having a limited amount of data. Here, data are considered to be limited if they contain less than 10,000 annotated particles.

The rest of the paper is organized as follows: In the first section, we briefly explain data sets and models employed for macromolecule identification and localization. In the proceeding section, we evaluate the model through various experiments. This is followed by presenting numerical and visual results. Finally, we discuss the limitations and future work of the research line.

## Materials and methodology

### Data set

**Fig. 2:**
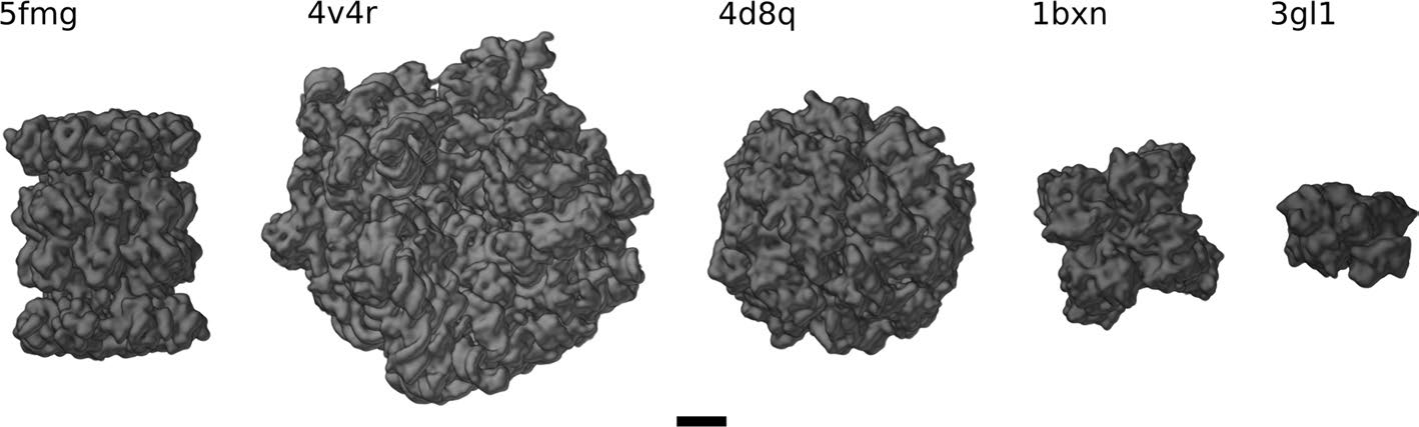
From Left to Right the density maps of the particles used in this paper are: Proteasome (5fmg), Ribosome (4v4r), Eukaryotic Chaperonin TRiC/CC (4d8q), Rubisco (1bxn), and ATPase domain of Ssb1 Chaperone (3gl1)

We employed two data sets to perform our experiments. The first is an experimental data set and is composed of four tomograms, each annotated for two macromolecules, namely 70S ribosomes from E. coli, and 20S proteasomes from T. acidophilum, respectively. Figure 2 illustrates the density maps of the particles used in our study.

The original voxel dimensions of the tomograms were $3712 \times 3712 \times 1392$ with a voxel size of 1.1 Å. For all experiments reported here, tomograms were down-sampled to $410 \times 410 \times 154$ voxels to yield an isotropic voxel size of 10 Å. Down-sampling is needed to increase the receptive field such that the neural network can see even large macromolecules in their full extent. In addition to down-sampling, a low-pass Gaussian filter [18] is applied to the tomograms. Tomograms were reconstructed from tilt series acquired in the angular range of $\pm 60^{\circ }$with a $3^{\circ }$ increment. The expert annotation was obtained by applying template matching followed by multi-ref classification [19] in STOPGAP [20]. Table 1 shows the distribution of the particle classes over all tomograms in the experimental data set. For more detail please see Additional file 1. The experimental data will be available for research purposes upon request and it will be made publicly available on EMPIAR database in near future.

**Table 1:** Distribution of proteasomes and ribosomes in the experimental data set

	T1	T2	T3	T4	Total
Proteasomes (5FMG)	327	314	323	281	1245
Ribosomes (4V4R)	196	176	206	168	746
Total	523	490	529	449	1991

The second data set consists of the 10 simulated tomograms that have been released for the SHREC’19 challenge by the University of Utrecht [10]. The data set contains 12 different molecules of different size and shape. The average number of molecules per tomogram is 208 per class. In order to have a fair comparison of the accuracy and $F_{1}$ scores between the two data sets, we selected three particles that cover large, medium-sized, and tiny macromolecules. The size of the simulated tomograms is $512 \times 512 \times 512$ voxels. No down-sampling or denoising was applied to the tomograms. Table 2 shows the number of particles per tomogram in the SHREC’19 data set.

**Table 2:** Distribution of macromolecular particles in the SHREC’19 data set

	T0	T1	T2	T3	T4	T5	T6	T7	T8	T9	Total
4D8Q	239	180	222	207	198	217	210	207	200	210	2090
1BXN	198	205	213	241	209	215	202	197	206	220	2106
3GL1	207	235	195	221	203	213	220	196	201	191	2082
Total	644	620	630	669	610	645	632	600	607	621	6278

### Method

**Fig. 3:**
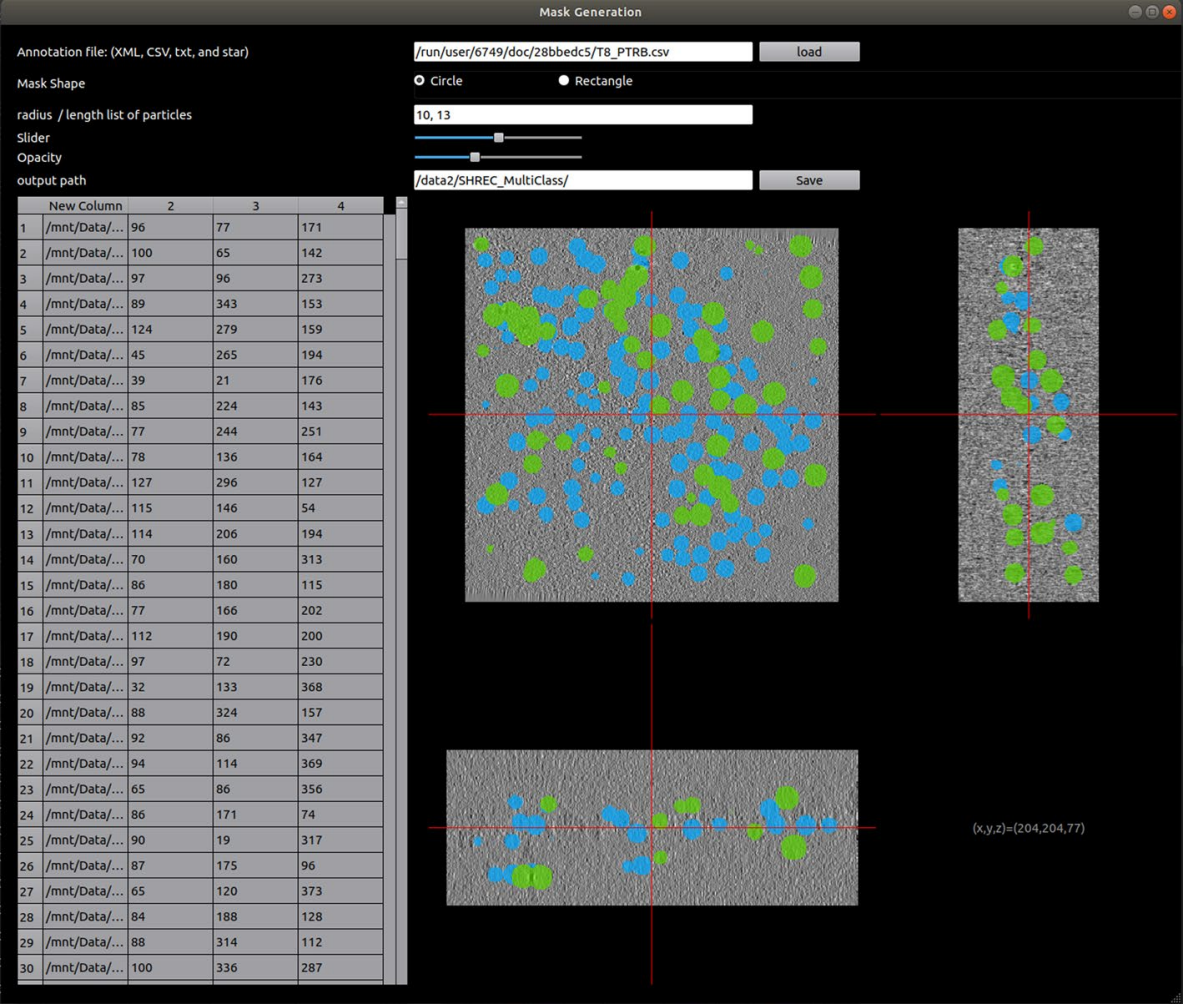
Mask generation

In order to train the networks, pairs of tomograms and ground truth labels are needed. In the simulated data, the exact particle locations are known while in the experimental data such coordinates are provided by an expert. Similar to Moebel et al. [2], we use sphere-shaped masks to generate ground truth labels, that is, 3D spheres enclosing each macromolecule instance at the respective location in the tomogram with a radius corresponding to the size of the target macromolecule. The main drawback of sphere-shape masks is that they add a certain amount of label noise to the ground truth. However, we selected this mask generation strategy in order to show the robustness of our model against label noise in the training data. Example of a generated mask using our developed GUI is shown in Fig. 3.

The first set of experiments is meant to evaluate the proposed method for binary and multi-class particle localization and identification. To do so, we generate training data sets for different numbers of classes. For the experimental data, we generate two binary and one multi-class subset, denoted as PT-BG, RB-BG, and PT-RB-BG, where PT, RB, and BG stand for Proteasome, Ribosome, and Background, respectively. As mentioned before, for the SHREC’19 data set, we use only three classes of macromolecule out of the available 12 classes. We selected 4D8Q, 1BXN, and 3GL1 as representatives of large, medium-sized, and tiny particle classes. Here, we refer to these binary and multi-class subsets as 4D8Q-BG, 1BXN-BG, 3GL1-BG, and 4D8Q-1BXN-3GL1-BG, respectively. We keep 17 % of the total particles for validation by applying 6-fold cross-validation on the training data; no data augmentation is used.

We chose DeepFinder^1^ as the model for numerical comparison as this can be considered state of the art. DeepFinder is based on the UNet structure that employs the encoder-decoder paradigm with skip connections. The encoder is a down-sampling network component employing max-pooling and convolution layers to extract general features of the samples while the decoder is an up-sampling component using the extracted features, skip connections and upconvolution to output high-resolution label maps. It consists of two down-/up-sampling layers in both components with filters of size $3 \times 3 \times 3$. The number of network parameters is $\sim 903$K. We train this network using all the aforementioned binary and multi-class data sets for macromolecule identification and localization.

**Fig. 4:**
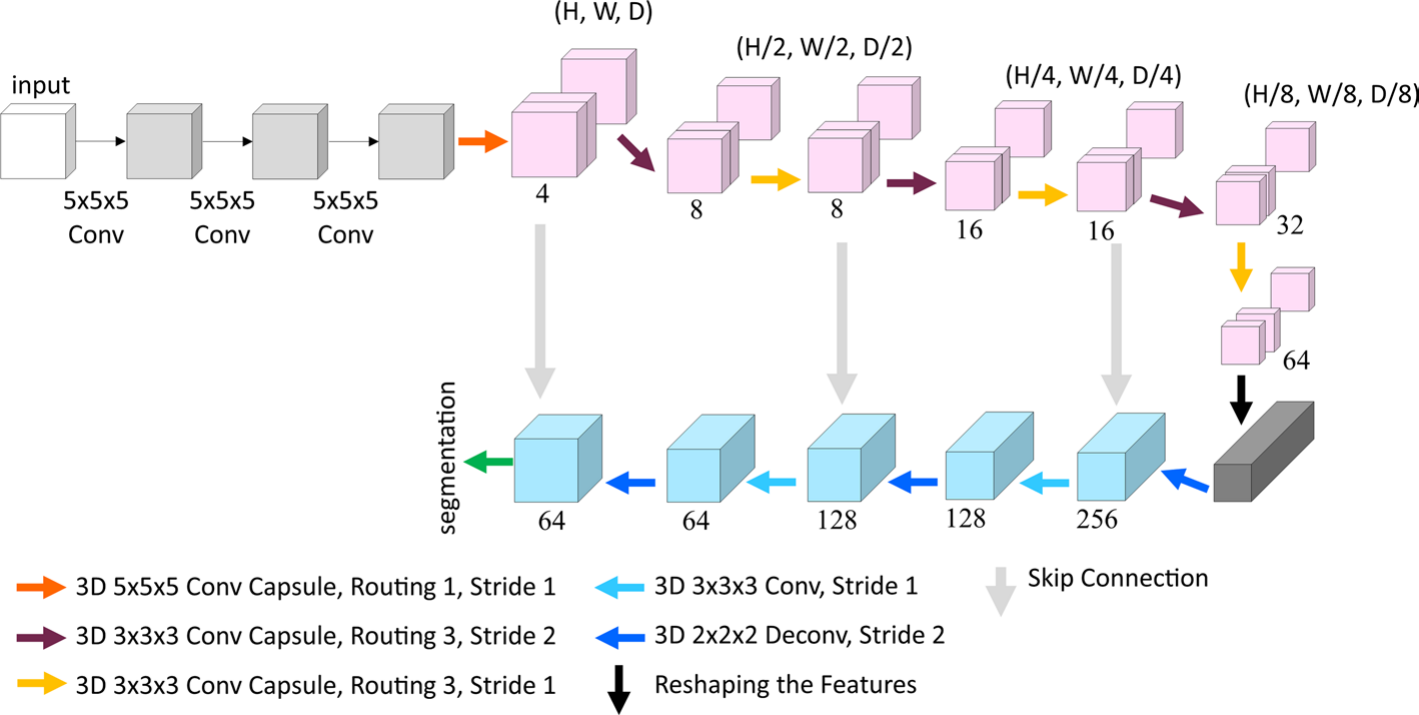
Block diagram of the 3D-UCaps network architecture. Numbers indicate capsule dimensions and channels at the encoder and decoder, respectively

Next, we employed the 3D-UCaps to define a network architecture for the same task, that is, macromolecule identification and localization. Figure 4 shows the block diagram of the network with more details about kernel sizes and number of layers.

Similar to DeepFinder, 3D-UCaps^2^ is based on a UNet structure with a 5-layer encoder-decoder component. However, 3D-UCaps is different in that it uses a 3D CapsNet in the encoder part instead of a classical CNN architecture. In addition, the network is initialized with a feature extractor component that converts voxel intensities to activities of local features so that it can be used by the primary capsules of the encoder. The 3D capsules in the encoder then extract the contextual information. The decoder benefits from skip connections and batch normalization layers. We set the size of the kernels in the feature extractor to be $5 \times 5 \times 5$ and the output to be a feature map of size $h \times w \times d \times 64$. These outputs are reshaped into grids of $h \times w \times d$, each representing a 64D feature vector. The convolutional layers of the decoder have filters of size $3 \times 3 \times 3$. The total number of trainable parameters is $\sim 3M$. It should be noted that an equivalent CNN architecture with the same amount of parameters would require a larger amount of data to be fully optimized. All training experiments were performed on an Nvidia Quadro RTX 6000 GPU. To present a reproducible work, our specific implementations of these methods and results are made publicly available^3^^,^^4^.

The main motivation to adopt this architecture is that CNN performance degrades when an image holds a slightly different orientation of the same object compared to the images used during training. In other words, in order to detect macromolecules from any viewpoint, we must have all those viewpoints within the training data because the prior knowledge of the geometrical relationships cannot be modeled in the network architecture. That is, a CNN-based model cannot capture the spatial relationships of the entities. This is mainly due to stacking layers on top of each other to build abstract context from low-level features. However, capsule-based architectures are robust toward geometrical transformations by encapsulating multiple layers within a capsule and capturing internal structures of the entities. As mentioned before, one challenge in particle identification using CET images is that particles can appear in many varying orientations. Since CapsNet is viewpoint-invariant, it can handle the identification problem requiring fewer varying viewpoints of the same particle.

CNNs use replicas of extracted features and max-pooling to output scalar feature detectors which are replaced by vector-output capsules and routing-by-agreement in CapsNet. Here, the learned features are still replicated across the space by having higher-level capsules covering larger regions of the image. However, unlike max-pooling, information about exact location of the particle is not discarded but place-coded. This is achieved using a routing algorithm that prevents exponential depth growth by choosing which capsules will be activated in the next layer. Capsules of each layer apply a set of learned filters to detect features and orientation of a particle. If the capsules agree strongly on particle existence, then the output is propagated to the most relevant capsules of the next layer. The route of activated capsules represents a low-level-to-high-level hierarchy of the particles. This allows to have architectures with deeper or higher number of down-/up-sampling layers. The features learned by CNNs contain only the information about existence and texture features aggregated through lower-level layers to the higher-level ones. This leads to the problem that CNNs do not generalize well to unseen geometrical changes of the input image. In CapsNets, fine-grained information is retained through learned features aggregated in higher-level layers. The outputs of each capsule is a high-dimensional vector encoding presence and orientation of the particles. In fact, CapsNets use the *non-linear squash function* to represent the probability that the particle is present. This function can be written as:

$$\begin{aligned} \varvec{v}_j = \frac{\Vert \varvec{s}_j \Vert ^2}{1 + \Vert \varvec{s}_j \Vert ^2}\frac{\varvec{s}_j}{\Vert \varvec{s}_j \Vert } \end{aligned}$$

Here, the total input and vector output of capsule *j* is indicated by $\varvec{s}_j$ and $\varvec{v}_j$, respectively. Let $\varvec{W}_{ij}$ be the weight matrix and $u_i$ be the vectors predicted by the layer below. Then, $\varvec{s}_j$ is calculated as a weighted sum by:

$$\begin{aligned} \varvec{s}_j = \sum _{i} c_{ij} \varvec{W}_{ij}\varvec{u}_i, \end{aligned}$$

where $c_{ij}$ are coupling coefficients.

It is also important to mention that CNNs have difficulty in generalizing to new viewpoints when trained on limited amount of data [13], while CapsNets convert intensities into vectors of instantiating parameters and then apply transformation matrices to overcome that problem. We modified the architecture slightly by replacing the dilated convolutions in the feature extractor with regular ones as the spreading out of the receptive field was deteriorating the result. The cross-entropy (CE) loss is replaced by joint Dice and CE loss to achieve a better classification performance. Also, instead of using Masked Mean-Squared Error for reconstruction loss, we used weighted cross-entropy that is reported to be more successful in case of having imbalanced samples [2].

## Experimental results and analysis

### Experiments

Hyper-parameters like batch size and optimizer were set to similar values for both networks. Other parameters like number of epochs mentioned here were defined based on the network convergence. The models were computationally trained with the ADAM optimizer algorithm with a learning rate of 0.0001. The batch size was set to 24 and the patch size to $64 \times 64 \times 64$ voxels. Due to the small size of the macromolecules within the large tomograms, there is a high class imbalance between the particle classes and the background class. Only around 1$\%$ of the voxels represent macromolecules while the rest are background voxels [2]. To address this problem, networks were trained using training patches extracted using the macromolecule locations. Networks were trained for 300 epochs with an early stopping criterion and it was always the case that an under-trained 3D-UCaps with early stopping converged in less than 300 epochs.

**Fig. 5:**
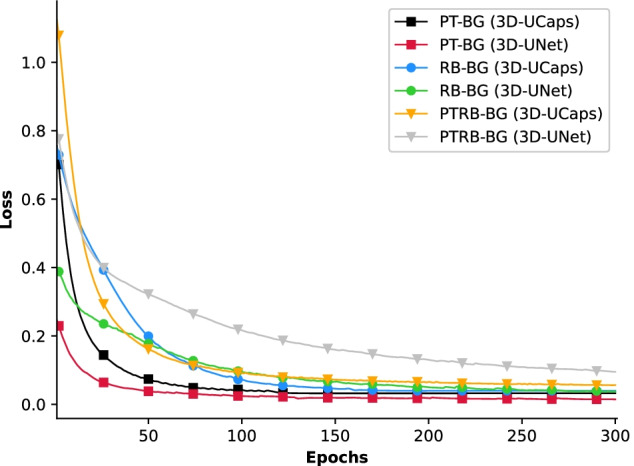
Learning curve for the 3D-UCaps and 3D-UNet network architectures using PT-BG, RB-BG, and PT-RB-BG of the experimental data set

Figure 5 depicts the learning curves for binary and multi-class training of 3D-UNet and 3D-UCaps on the real data. The two models exhibited similar rate of decay, however, the 3D-UNet converged to a lower average loss for PT-BG data set than the 3D-UCaps (0.03 vs. 0.06). For the PT-RB-BG dataset, 3D-UCaps achieved a significantly lower average loss (0.12 vs. 0.20). The average loss is comparable when training is done using the RB-BG data set. In this case, 3D-UCaps and 3D-UNet were optimized with loss values of 0.10 and 0.09, respectively. The learning curve represents the segmentation quality and generalization capabilities of each model while the $F_1$ scores represented in the next section quantify the localization performance.

In order to assess the quality of a prediction, the $F_1$ score is commonly used. It is based on two other measures, Precision and Recall. Precision is the ratio of correctly predicted positive observations and total predicted positive observations. It helps to understand how often the model correctly predicts positive. This metric is important when the cost of predicting a false positive is high. Recall, on the other hand, is an important factor when the cost of predicting false negatives is high. In fact, Recall presents the ratio of correctly predicted positive observations and all observations in the positive class. In order to have a correct interpretation of the performance, $F_1$ score is introduced as a metric that considers both Precision and Recall. This score is the weighted average of Precision and Recall and takes both false positives and false negatives into account. Thus, it is reliable when we have an imbalanced class distribution. These metrics are defined as follows:

$$\begin{aligned} \text {Pr} = \frac{TP}{TP+FP}, \quad \text {Re} = \frac{TP}{TP+FN}, \quad \text {F}_{1} = 2 \times \frac{\text {Pr} \times \text {Re}}{\text {Pr} + \text {Re}} \end{aligned}$$

#### 3D-UNet

DeepFinder (3D-UNet) is trained for multi-class and binary macromolecule identification. Moebel et al. used four different data sets [2]. The fourth data set had the same number of tomograms and the lowest number of annotations and classes as our real data set. As a result, we followed almost the same training scheme as Moebel et al. Table 3 compares $F_{1}$ score, Precision and Recall for training the networks under multi-class and binary macromolecule identification using real and simulated data. Results are presented for both validation and test data. A large gap between the validation and test results suggests an over-fitting problem.

**Table 3:** 3D-UNet performance comparison for $F_{1}$ score, Precision (Pr.), Recall (Re.) with respect to the number of classes

	Validation			Test		
	$F_{1}$	Pr.	Re.	$F_{1}$	Pr.	Re.
*Real data*						
PT-BG	0.91	0.89	0.92	**0.79**	0.77	0.81
RB-BG	0.73	0.64	0.85	0.60	0.54	0.67
PT-RB-BG	0.73	0.70	0.77	0.64	0.61	0.68
*SHREC’19*						
4D8Q-BG	**0.93**	0.89	0.97	**0.93**	0.90	0.96
1BXN-BG	0.83	0.73	0.95	0.82	0.71	0.98
3GL1-BG$^{\dagger }$	0.50	0.39	0.71	0.29	0.22	0.42
4D8Q-1BXN-3GL1-BG	0.80	0.74	0.87	0.69	0.64	0.74

$^{\dagger }$Training patch size is $32 \times 32 \times 32$. More details is in “Case Study” section

#### Training 3D-UCaps

Similarly, 3D-UCaps is trained on real and simulated data under multi-class and binary classification scenarios. In general, the multi-class scenario provided a better performance, which is in line with the available results in the literature [2, 3]. Similar to 3D-UNet, we present the $F_{1}$ score, Precision and Recall for training 3D-UCaps in Table 4. Training is done with similar hyper-parameters mentioned in the Experiments section.

**Table 4:** **3D-UCaps** performance comparison for $F_{1}$ score, Precision (Pr.), Recall (Re.) with respect to the number of classes

	Validation			Test		
	$F_{1}$	Pr.	Re.	$F_{1}$	Pr.	Re.
*Real data*						
PT-BG	**0.93**	0.95	0.91	0.74	0.77	0.71
RB-BG	**0.83**	0.89	0.77	**0.68**	0.76	0.62
PT-RB-BG	**0.93**	0.96	0.90	**0.91**	0.95	0.87
*SHREC’19*						
4D8Q-BG	0.92	0.95	0.89	0.92	0.95	0.88
1BXN-BG	**0.88**	0.90	0.85	**0.86**	0.89	0.82
3GL1-BG$^{\dagger }$	**0.51**	0.55	0.47	**0.32**	0.26	0.41
4D8Q-1BXN-3GL1-BG	**0.86**	0.91	0.81	**0.71**	0.79	0.64

$^{\dagger }$Training patch size is $32 \times 32 \times 32$. More details is in “Case Study” section

#### Comparison and analysis

From Tables 3 and 4, it can be seen that both models show a robust performance regardless of type of data (real or simulated) being used for training. However, regarding the difference between the $F_1$ scores of validation and test sets, we can observe that the experimental data show larger performance degradation than the artificial data. This could be explained by incorrect labels generated during annotation and lower signal-to-noise ratio of the experimental data set. In particular, there were differences of 0.19 and 0.13 between $F_1$ scores of validation and test sets of PT-BG data set for 3D-UNet and 3D-UCaps, respectively.

**Fig. 6:**
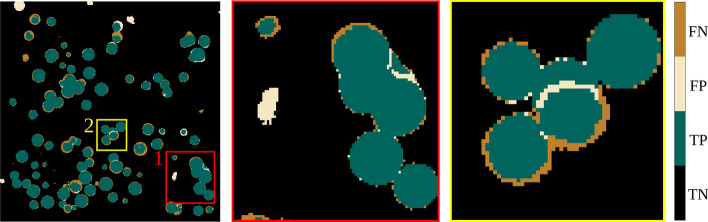
Representation of a predicted mask overlapped with a ground truth mask (**3D-UCaps**)

**Fig. 7:**
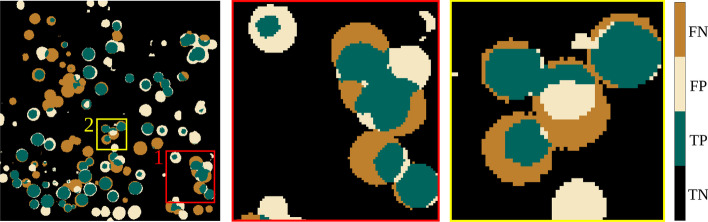
Representation of a predicted mask overlapped with a ground truth mask (**3D-UNet**)

A close $F_1$ score of validation and test data demonstrates a successful learning procedure that relies on general features such as geometrical shapes and textures. In general, we observed that the $F_1$ scores between 3D-UNet and 3D-UCaps were close. However, we can see that the slightly higher $F_1$ score of 3D-UCaps often coincides with a higher Precision score (Bold in Table 4). This can be seen more prominently when we look at pixel-wise predictions on the individual tomograms. To support this observation, we overlapped a representative predicted mask with a ground truth mask to evaluate the True Positives (TP), False Positives (FP), False Negatives (FN), and True Negatives (TN) using pseudo colors. We noticed that the 3D-UCaps predicted more TP pixels than the 3D-UNet (see Figs. 6 and 7, subregion 2). The 3D-UNet, in contrast, predicted more FN pixels which causes a reduction of the overall $F_1$ score (see Figs. 6 and 7, subregion 1). By further investigation of the predicted masks, we also observed that in general the mask region was over-predicted in the 3D-UNet (see Fig. 7, rings of FP pixels), whereas the 3D-UCaps predicted a subregion of the ground truth mask (see Fig. 6, rings of FN pixels). We speculate that the better precision of 3D-UCaps compared to 3D-UNet is due to the capsule network employed in the encoder.

**Fig. 8:**
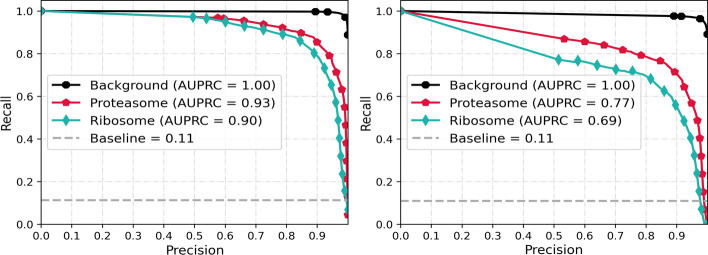
Precision-recall curve, left: **3D-UCaps**, right : **3D-UNet**

The aforementioned interpretation is justified by illustrating the area under the precision-recall curve (AUPRC) in Fig. 8. AUPRC is another performance metric for imbalanced data. Here the baseline in AUPRC curve is defined as the fraction of positive pixels over total number of pixels.

### Visualization

**Fig. 9:**
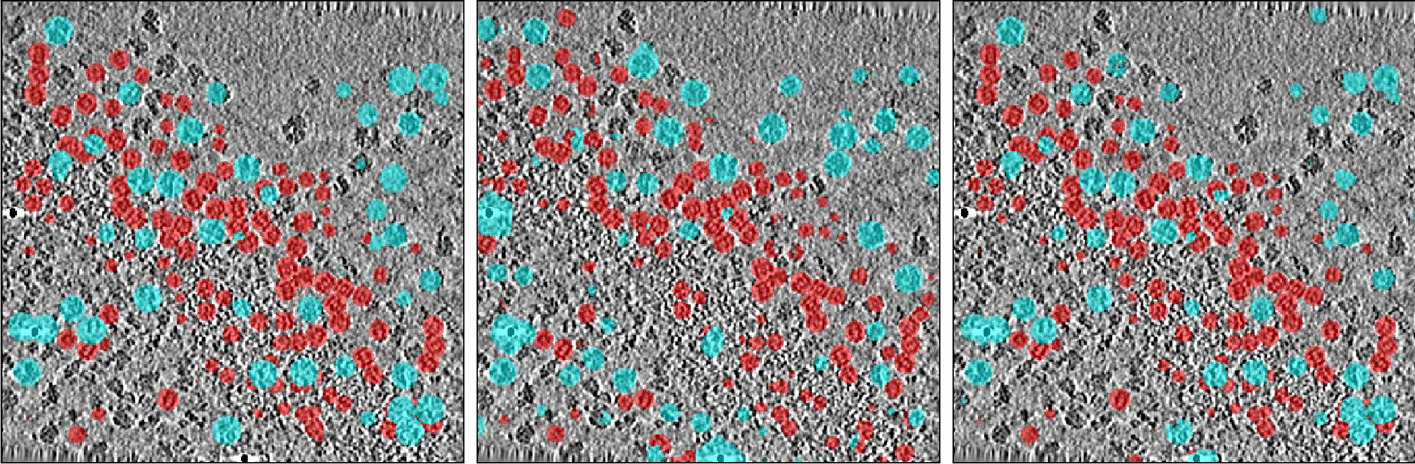
Visualization of the label maps for the multi-class scenario; From left to right: ground truth, 3D-UNet, and 3D-UCaps results. Red spheres indicate proteasome macromolecules while blue represents ribosomes

Figure 9 presents a visualization of the results for the test tomogram of the multi-class scenario in experimental set. Visualization on the validation data on other scenarios and data set are provided within the material of the code implementation. Prediction is performed on the patches with an overlap of 25 voxels and stitched afterward to generate the whole tomogram. It is important to note that naturally, the stitching process produces sharp edges and other artifacts around the borders that is addressed by averaging on those regions.

### Architecture evaluation

**Fig. 10:**
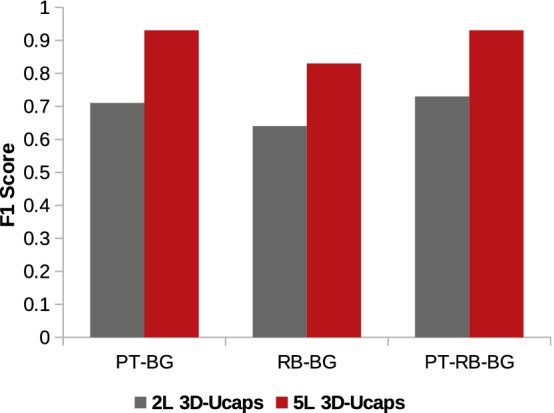
Comparison of the $F_1$ scores between the 5- and 2-layers 3D-UCaps

In this section, we analyse the model performance by applying slight changes to the 3D-UCaps architecture. We study the effect of reducing the number of layers on the performance. Moebel et al. [2] report that using more than two down-sampling stages does not improve the result. Originally, 3D-UCaps has 5 layers. In order to study such effect within the 3D-UCaps structure, we discarded 3 layers from the encoder and decoder. Then, we used the set of bench-marked parameters to train the network on the experimental data. Figure 10 compares the results of the 5- and 2-layers 3D-UCaps using the validation set of the experimental data. Our experimental results show that the 5-layer network always performs better than the 2-layer network.

### Ablation study

Originally, 3D-UCaps uses dilated convolution in the feature extractor component. Dilated convolution modifies convolution kernels by defining a spacing between the values in the kernel. The main reason behind using dilated convolution is having a larger receptive field without extra computational cost. We conducted two experiments to study the effect of dilated convolution by replacing the dilated convolutions with regular ones. The enlarging behaviour of the dilated kernels appeared to be destructive by reducing the $F_1$ scores in multiclass experimental data from 91% to 86%. Also, enlarging the kernel size of convolution kernels in the encoder from $3 \times 3 \times 3$ to $5 \times 5 \times 5$ degraded the performance from 91% to 83%. The deterioration of the result is more dramatic in the case of binary classification of small particles.

### Case study

**Fig. 11:**
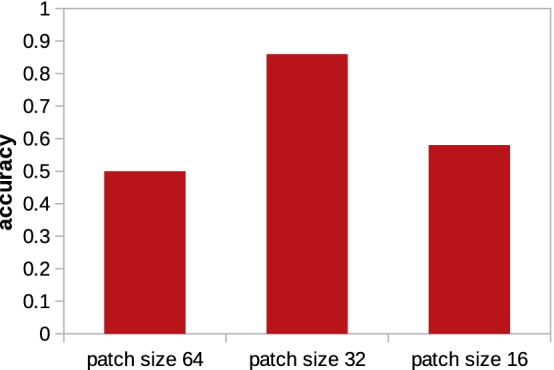
Comparison of the $F_1$ scores for training 3D-UCaps using patches of size $64 \times 64 \times 64$, $32 \times 32 \times 32$, and $16 \times 16 \times 16$

We also studied the performance of the models considering localization of tiny particles like 3GL1. This is particularly of high importance as it is reported that localization of particles with small radius in binary classification scenarios is extremely challenging [2]. We trained the 3D-UCaps using patches of size $64^3$ and $32^3$ where the former is the reasonable size for a particle with radius 13 located at the center of the patch. We observed that choosing an appropriate patch size based on the probe particle is playing an important role. Reducing the patch size from cubes of $64^3$ voxels to $32^3$ improved the performance considerably. However, the patch size reduction has a limitation considering the kernel sizes of the network. Once, we decreased the patch dimension to $16^3$, the network performance drops again due to having large kernels. Reducing the patch size for tiny particles helps reducing the background noise fed to the system at the training time. Figure 11 compares the results of 3D-UCaps with respect to training patches of size 64, 32, and 16.

### Performance

As mentioned before, all training procedures were achieved using an Nvidia Quadro RTX 6000 GPU, by using Cuda 11 and cuDNN 8. In Table 5, we display the average training and inference time required to train and test the models using the simulated data and the set of hyper-parameters reported in the Experiments section.

**Table 5:** **3D-UCaps** performance comparison for $F_{1}$ score, Precision (Pr.), Recall (Re.) with respect to the number of classes. **h** and **m** stand for hour and minutes, respectively

	3D-UNet		3D-UCaps	
	Training (h)	Test (m)	Training (h)	Test (m)
Multi-class	20	6	48	14
Binary	7	6	15	11

## Discussion

In this study, a new deep learning approach based on capsule networks is employed for macromolecule segmentation in CET. The network consists of a U-Net architecture where the encoder component is a 3D CapsNet while the decoder is based on the conventional 3D CNN applying the volumetric segmentation. Also, a feature extractor is used for initializing the primary capsules of the encoder. The performance of the network is better than the 3D-UNet in multi-class scenarios and the results of binary classification is also slightly improved.

While the amount of data used for training both models were similar, the number of trainable parameters in 3D-UCaps is larger than that of 3D-UNet. This means we achieved a deeper architecture that can be trained with limited amount of data while preserving the performance in terms of accuracy and $F_1$ score. Our ablation study supports the idea of having higher number of layers is possible by using Capsule layers in the encoder component. Having deeper architectures is significant because low-level extracted features merely reveal abstract properties like edges about the detected objects. However, it is the high-level extracted features that present more complex concepts like geometrical shapes, locality, and hierarchy of the object with respect to its neighborhood region. Another advantage of the 3D-UCaps network are the small differences between validation and test results that show that no overfitting occurred. As Capsule layers extract both the existence probability and the orientation of the object, such architectures are of high importance in downstream tasks such as macromolecule reconstruction or submolecular unit detection.

A slight drawback of the 3D-UCaps model is the high computational time for training, which is approximately twice as high compared to 3D-UNet. However, the inference time is reasonable once the pre-trained model is available.

Future work includes reducing the model complexity and computational cost of the training, improving segmentation result on the test data, as well as studying other hybrid architectures that use CapsNet for tasks where extracted orientation features plays an important role.

## Supplementary Information

**Additional file 1**. Supplementary information on the experimental data set.

## Data Availability

The experimental data will be available for research purposes upon request. The interested readers can contact Sagar Khavnekar via khavnekar@biochem.mpg.de. Additionally, the experimental data will be made publicly available on EMPIAR database in near future.

## Footnotes

1 https://gitlab.inria.fr/serpico/deep-finder.

2 https://github.com/VinAIResearch/3D-UCaps.

3 https://github.com/noushinha/DeepET.

4 https://github.com/noushinha/3D-UCaps.
