# Supplementary material for: Volumetric macromolecule identification in cryo-electron tomograms using capsule networks
Source: BMC Bioinformatics. 2022 Aug 30;23:360. doi: 10.1186/s12859-022-04901-w (PMC9429335; doi:10.1186/s12859-022-04901-w)
Supplement: Supplementary file 1 — Additional file 1. Supplementary information on the experimental data set. [file 12859_2022_4901_MOESM1_ESM.pdf]

Additional file 1 — Supplementary information on experimental data set

For the mixed ribsome and proteasome dataset, purified E. coli 70S ribosomes and T. acidophilum 20S proteasomes at equimolar concentration (3.0 mg/mL and 1.5 mg/mL, respectively) were mixed with 10 nm gold fiducials. 4.5 ml of the mixture were applied onto a glow-discharged 200 mesh Quantifoil R2/1 copper grid. Samples were vitrified in a liquid ethane/propane mixture using a Vitrobot Mark IV (Thermo Fisher Scientific) set to 4°C and 100% humidity. Settings: blot f orce = 20 and blot time = 4.5 second. Before loading into the Transmission Electron Microscope (TEM), all grids were clipped in Autogrids.

The Cryo-ET data was acquired using a Thermo Scientific Titan Krios equipped with Gatan Bioquantum energy filter and K2 summit Direct Electron Detector. Tilt-series were collected with a dose-symmetric tilt scheme usingSerialEM 3.8 with automated stigmation, coma-free alignment, and coma versus image-shift compensation [21]. The tilt range was ±60° with 3° increments. Tilt images were preceded by tracking and autofocus and were tracked after acquisition. The target focus was changed per tilt-series in steps of 0.25 µm over a range of -1.25 µm to -2.75 µm. Images were acquired in counting mode with a calibrated pixel size of 1.1 ˚A and a total dose of 3 e−/ ˚A2 over ten frames. Tilt-series preprocessing and tomogram reconstruction: The data was preprocessed using TOMOgram MANager (TOMOMAN) [22]. In case of K2 summit data acquisition, MOTIONCOR2 [23] was used for motion correction. The tilt-series were corrected for dose-exposure using MATLAB (MathWorks) scripts adapted for tilt series [REF]. Defocus was estimated using CTFFIND4 [24]. Tilt series were aligned using fiducial based alignment in IMOD [25]. Gold beads were automatically selected and tracked. The resulting fiducial model was corrected manually in all cases where automatic selection and tracking failed. Initial tomograms without CTF correction were reconstructed by weighted back projection (WBP) at 8x binning and used for template matching. For subtomogram averaging, tomograms were reconstructed with 3D-CTF correction using NovaCTF [26] with phase-flip correction, astigmatism correction using 15 nanometer slab thickness. Tomograms were binned 2x, 4x, and 8x using FourierCrop3D [26].

Initial particle positions for both 20S Proteasome and 70S Ribosomes were determined using noise correlation template matching approach implemented in STOPGAP[20]. PDB entries, 5FMG for 20S Proteasome and 4V4R for 70S Ribosomes were used to generate templates using molmap [27] command in Chimera [28]. Subsequent subtomogram averaging and classification were performed using STOPGAP [20]. Classification was performed using simulated annealing stochastic hill climbing multi reference alignment as described before [29].
